# Reception of the American Medical Association at Independence Hall, Philadelphia, May 2, 1855

**Published:** 1855-07

**Authors:** 


*Reception of the American Medical Association at Independence Hall, Philadelphia, May* 2, 1855.

A full account of the speeches, etc., on this interesting occasion, was given in our last, but we are none the less indebted to the unknown hand which has favored us with this neat little pamphlet.

